# Post‐Mortem Community Surveillance of COVID‐19: Implementation and Evaluation of a Pilot System in the Funeral Sector in England, UK, January 2021 to February 2022

**DOI:** 10.1111/irv.70116

**Published:** 2025-06-10

**Authors:** Hannah E. Emmett, Jennifer Hall, Harriet H. Webster, Abigail Izzard, Anika Singanayagam, Maria Zambon, Gavin Dabrera

**Affiliations:** ^1^ UK Field Epidemiology Training Programme UK Health Security Agency London UK; ^2^ COVID‐19 Vaccines and Epidemiology Division UK Health Security Agency London UK; ^3^ North London Health Protection Team UK Health Security Agency London UK; ^4^ EGA Institute for Women's Health University College London London UK; ^5^ COVID‐19 National Virology Cell UK Health Security Agency London UK

**Keywords:** COVID‐19, evaluation, feasibility, post‐mortem, SARS‐CoV‐2, surveillance

## Abstract

**Background:**

Early in the COVID‐19 pandemic, due to limited testing, a potential gap in capturing SARS‐CoV‐2‐positive community deaths was identified. Post‐mortem testing for respiratory viruses had never been implemented in the United Kingdom.

**Aim:**

Through implementing and evaluating a pilot, we aimed to establish feasibility and acceptability of post‐mortem SARS‐CoV‐2 surveillance using funeral directors (FDs) to capture ‘missed’ COVID‐19 community deaths.

**Methods:**

Between January 2021 and February 2022, four FDs took upper respiratory tract samples from eligible people who died outside hospital. We tested for SARS‐CoV‐2 and other respiratory viruses using reverse transcription‐polymerase chain reaction and matched results to the national COVID‐19 mortality dataset. We evaluated the pilot for acceptability, data completeness and timeliness, and simplicity, using semi‐structured interviews, a questionnaire, and data audit.

**Results:**

Two thousand eight hundred sixty‐five deaths were handled by FDs: 998 were assessed for eligibility, 342 were eligible 81 were tested. Eight were SARS‐CoV‐2‐positive, of which three were not identified by ante‐mortem clinical testing. The programme was acceptable in principle to FDs and families, but FDs' participation was limited by the burden of legal requirements and existing workloads. Families' willingness to consent fluctuated (monthly consent rate 4–83%, overall 30%); fewer consented when overall cases were low. Completeness and timeliness of data was good. FDs judged the programme simple.

**Conclusion:**

The pilot established feasibility and demonstrated, even with small numbers, the ability to detect ‘missed’ deaths. There were significant obstacles to implementation. Alternative settings for taking specimens are being explored instead to address this gap in national surveillance.

## 1 Introduction

Early in the COVID‐19 pandemic, concerns were raised that community deaths either with or from SARS‐CoV‐2 infection were inadequately captured. By 5 July 2020, there had been 13,186 deaths in England where COVID‐19 was documented on the medical certificate of cause of death but which did not have a confirmatory test, either ante or post‐mortem. There was also emerging evidence that COVID‐19 may be linked to thrombotic and cardiovascular events [1]. This led to concerns that deaths where COVID‐19 may have been a contributing factor would not be identified as such.

Between January and May 2020 in the UK, testing for SARS‐CoV‐2 only occurred as part of a clinical pathway in secondary care, or in the community for key workers, and was not routinely available to the general public [2]. This is not dissimilar to testing for other respiratory viruses outside the pandemic context. From May 2020, community polymerase chain reaction (PCR) testing for symptomatic individuals became freely available, and capacity for this increased over the course of the pandemic. From November 2020, lateral flow device tests became freely available to the public. Free mass community testing in England ended in April 2022 [3, 4].

Surveillance using post‐mortem testing for respiratory pathogens had been considered in England previously but had not been trialled. Although the UK has a robust sentinel respiratory virus surveillance system, most community deaths are not tested for respiratory viruses ante‐mortem. A post‐mortem surveillance capacity could add to abilities to detect and respond to emerging viral threats, and to understand the burden of respiratory viruses in all‐cause mortality. Further, a report following the 2009 H1N1 influenza pandemic suggested there were gaps in national surveillance; the importance of strengthening national virological surveillance for respiratory viruses had therefore been on the agenda for some time [5].

Testing for SARS‐CoV‐2 using reverse transcription‐PCR (RT‐PCR) on upper respiratory tract (URT) samples taken within 7 days of death was shown to have a sensitivity of 96.8% if the individual had tested positive within the 7 days prior to death [6]. Feasibility of post‐mortem testing for seasonal respiratory viruses, including influenza A, respiratory syncytial virus, rhinovirus, and coronavirus, had been demonstrated by Navascués et al. [7]. Case reports from Germany and a study in Zambia found SARS‐CoV‐2 to be detectable in URT specimens in the first week and 48 h post‐mortem respectively [8, 9].

We conceptualised a voluntary, sentinel, post‐mortem surveillance programme for COVID‐19 from recent community deaths using the funeral sector, with the aim of better understanding COVID‐19‐related mortality in England outside of hospital settings. We aimed to assess the feasibility of such a system using a pilot, to identify and explore the barriers to implementation. This paper aims to describe the development and implementation of the pilot programme, and to evaluate it, in order to understand the feasibility of such a system and make recommendations for future surveillance of SARS‐CoV‐2 and other respiratory viruses.

## 2 Methods

### 2.1 Pilot Implementation

We consulted widely on the development of the programme: (1) within the national COVID‐19 public health response structure to establish its potential value, (2) with regional public health teams and emergency preparedness bodies to understand acceptability and interest, (3) with governmental bodies to establish its legal basis, (4) with professional membership organisations representing pathology and the funeral industry to understand acceptability to the sector, and (5) with representative organisations of major faith groups.

We chose to recruit funeral directors (FDs), organisations which manage funerals, in the Midlands area of England as this region had the highest all‐cause mortality rate in England at the time of the development of the pilot [10]. We recruited FDs by putting out an open call through a membership organisation of the funeral sector.

We worked with the Human Tissue Authority (HTA) to ensure the protocol for the surveillance pilot met legal requirements. The HTA regulates organisations which remove, store, and use human organs and tissue for research, medical treatment, post‐mortem examination, education and training, and display in public. We supported the recruited funeral directors to meet the legal requirements to obtain the ‘removal licence’ from the HTA to enable them to participate in the programme. The licence covers removal of tissue from the body of the deceased, in this case cells from the upper respiratory tract^1^ [11].

The population of interest was adults aged over 18, resident in England, who died in the community. The surveillance testing definition limited this to those who could have a URT swab taken within five (later seven) days of death, and where consent had been given. It excluded deaths which had occurred in hospital, as these individuals were more likely to have been tested clinically ante‐mortem; deaths under coronial jurisdiction,^2^ as post‐mortem testing would have required consent from the relevant coroner; and deaths where the body had undergone embalming, as this was likely to affect the validity of the test. Consent was sought from the person in the highest available ‘qualifying relationship’ to the deceased, in the hierarchy laid out in the Human Tissue Act 2004 [11].

FDs collected data using specimen request forms, relative consent forms, and information logs. These data sources contained personal identifiable information (PII) of the eligible deceased (information log) and their consenting relatives (specimen request form, relative consent form). The information log was used to document the deceased's pathway through the protocol: eligibility assessment, obtaining consent, taking the specimen, and posting the specimen.

**FIGURE 1:**
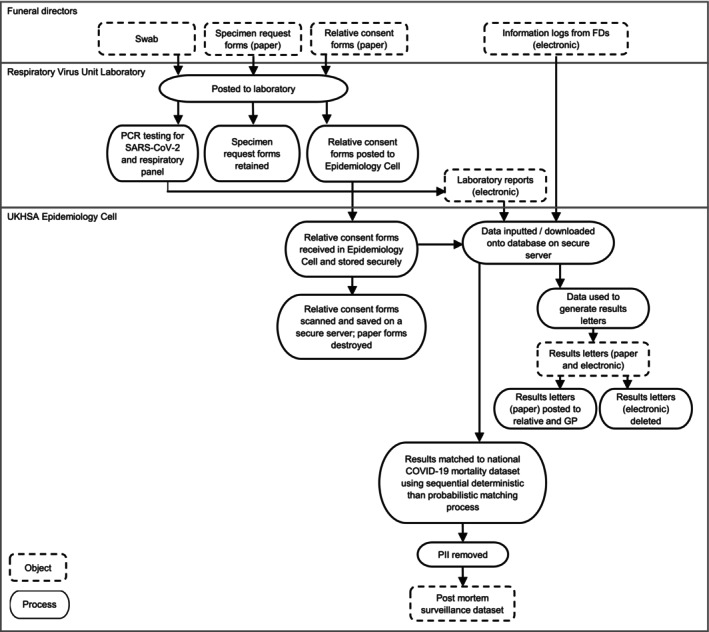
Pilot post‐mortem surveillance system process diagram.

FDs sent the URT specimen and the consent and specimen request forms to the national respiratory virus reference laboratory where specimens were tested using RT‐PCR for SARS‐CoV‐2, influenza, respiratory syncytial virus, and human metapneumovirus [12, 13, 14]. We extracted the test results from the laboratory information system into the post‐mortem surveillance database, created for this pilot. We linked the results to the national COVID‐19 mortality dataset using a sequential matching process to establish whether the deaths had been previously documented as COVID‐19‐related. We undertook deterministic matching: first on forename, surname, date of birth, and then on forename, surname, date of death. For results which did not match deterministically, we undertook probabilistic matching using soundexes (phonetic algorithms for indexing names to account for minor spelling differences) [15]: on soundex forename, exact surname, date of birth, then exact forename, soundex surname, date of birth, and finally soundex forename, soundex surname, date of birth. Where matched, whether the death occurred within 28 or 60 days of the latest ante‐mortem SARS‐CoV‐2‐positive result was included in the post‐mortem surveillance dataset (Figure 1).

### 2.2 Pilot Evaluation

We evaluated the pilot in terms of five system attributes, using quantitative and qualitative methods [16, 17, 18].

We evaluated acceptability to the funeral sector using a series of semi‐structured interviews with participating FDs during and at the end of the pilot. Topics included embedding the surveillance system into their existing work patterns, perception of the system amongst staff, barriers to implementation, willingness of families to consent, and support required from UKHSA. We used a short questionnaire for non‐participating FDs (those which had initially expressed an interest in participating but did not go on to do so), including questions on barriers to participation, and what factors may have facilitated participation. We evaluated acceptability to the HTA using a structured interview, and acceptability to the public using an audit of the data received via the information log.

We evaluated data quality through an audit of the completeness and timeliness of data submitted by participating FDs. Although not an objective of the pilot, we described the time taken from receipt of specimen to receipt of test results.

We evaluated the simplicity and flexibility from the perspective of the funeral sector through semi‐structured interviews with FDs and reviewing the timeline of changes made throughout the pilot.

Though it was not a stated aim of the pilot, we considered its potential usefulness through descriptive analysis of the data obtained.

Ethical approval for the pilot surveillance programme was obtained from the Public Health England Research, Ethics, and Governance Group.

## 3 Results

### 3.1 Data Summary

Following an open call in August 2020, seven FDs expressed an interest of which three went on to participate. A second call to expand the pilot in May 2021 resulted in 12 expressions of interest and one FD which went on to participate.

The pilot ran from January 2021 to February 2022. FD participation was staggered, with between one and four FDs participating throughout. Start and end months were as follows: (1) January 2021 to January 2022, (2) February 2021 to February 2022, (3) March 2021 to February 2022, (4) October 2021 to February 2022.

**FIGURE 2:**
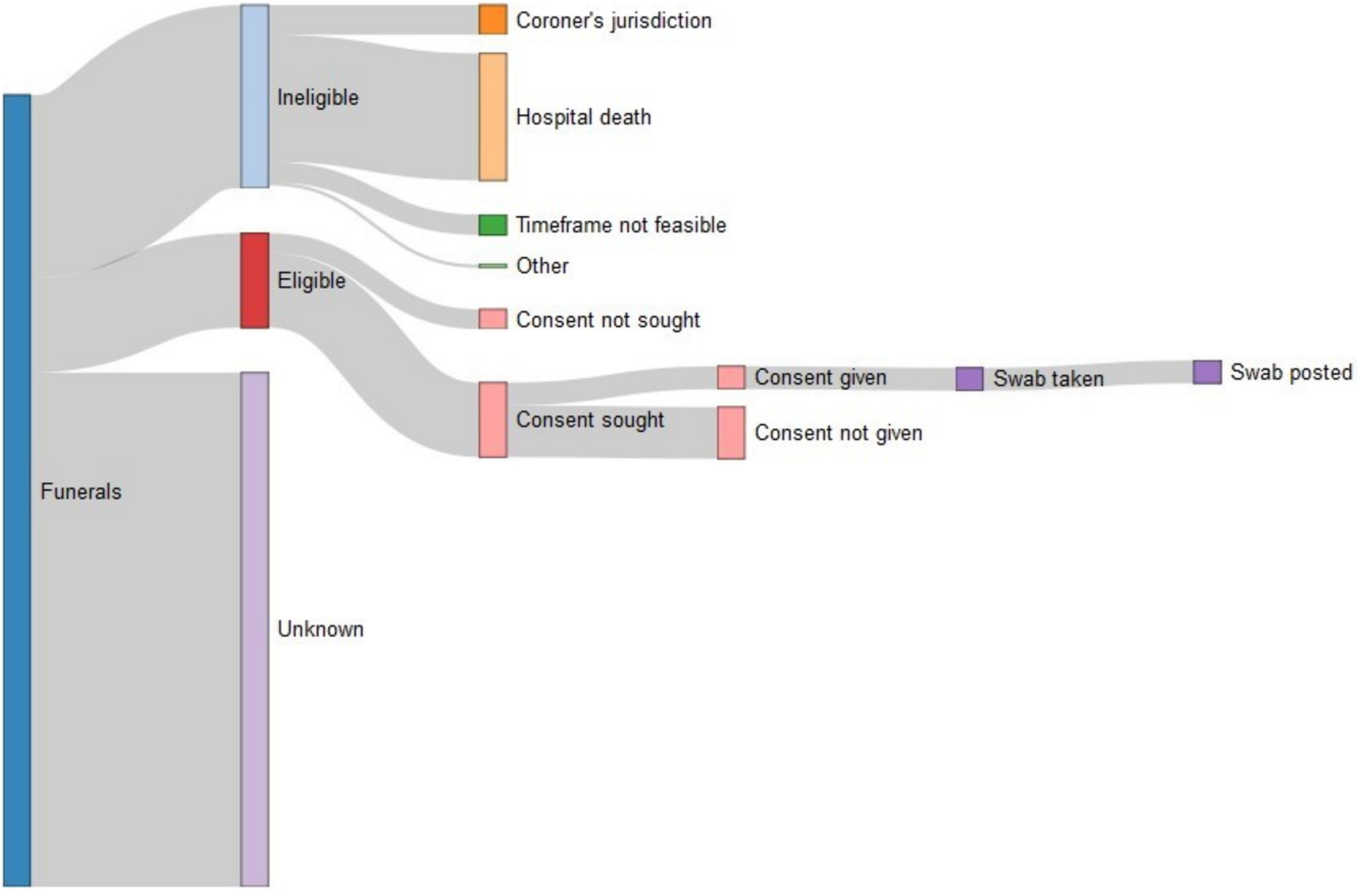
Sankey plot showing completeness of data flows of deceased individuals, represented as funerals, through the pilot system, January 2021 to February 2022.

In 14 months, participating FDs managed 2865 deaths of which 998 (34.8%) were assessed for eligibility. Three hundred forty‐two (34.3%) were considered eligible, of which 271 (79.2%) were asked for consent and from which 81 (23.7%) URT specimens were received. Eight (9.8%) tested positive for SARS‐CoV‐2 (Figure 2). Monthly numbers of specimens received were highest in February, March, and October 2021. A specimen was taken in all instances where consent was given. The proportions of relatives who gave consent for sampling were highest in January to April 2021 (69–73%) and lowest in December 2021 (4%) (Table 1).

**TABLE 1:** Number and percentage of eligible deceased, specimens taken, and SARS‐CoV‐2 positive results by month, January 2021 to February 2022 inclusive.

Month	Eligible deceased (*n*)	Specimens taken (*n*)	Specimens taken (%)	SARS‐CoV‐2 positive (*n*)	SARS‐CoV‐2 positive (%)
Jan‐21	12	10	83.3	3	30.0
Feb‐21	13	10	76.9	1	10.0
Mar‐21	13	9	69.2	0	0.0
Apr‐21	9	7	77.8	0	0.0
May‐21	16	3	18.8	0	0.0
Jun‐21	23	7	30.4	0	0.0
Jul‐21	27	3	11.1	0	0.0
Aug‐21	39	6	15.4	0	0.0
Sep‐21	38	13	34.2	3	23.1
Oct‐21	35	5	14.3	0	0.0
Nov‐21	44	6	13.6	1	16.7
Dec‐21	31	1	3.2	0	0.0
Jan‐22	25	1	4.0	0	0.0
Feb‐22	17	0	0.0	0	0.0
Total	342	81	23.7	8	9.9

When matched to the national COVID‐19 mortality dataset, three of the eight positive post‐mortem specimens were in individuals who had not been identified as a death within 28 days of a positive SARS‐CoV‐2 result. Of the 73 negative post‐mortem specimens, none had tested positive in the 28 days ante‐mortem.

None of the deceased tested positive for influenza, RSV, or metapneumovirus. This was in keeping with circulating respiratory infections during the pilot period [19].

### 3.2 Acceptability

Participating FDs reported that the overall concept of the pilot was acceptable, including carrying out sensitive conversations and taking specimens. However, they reported difficulty in managing the full range of activities required for the pilot, including assessing and documenting eligibility, alongside their existing workload. In some FD sites there was a difference in willingness to participate between management staff, who were keen to participate, and front of house staff, who reported insufficient capacity.

Of the 15 FDs which initially expressed an interest in the pilot but did not go on to participate, four reported that staffing shortages, including caused by COVID‐19, meant they could not commit to additional work. Others did not give a reason for not continuing with the registration process, and were lost to follow up.

Three non‐participating FDs responded to an anonymous multiple choice questionnaire, in which they reported barriers to participation. When asked which factors affected their decision not to join the pilot, ‘the resources required to carry out the work’ and ‘the training requirements of the pilot programme’ were reported by two FDs (67%). ‘The length of the application form for the HTA licence’, ‘the questions in the application form for the HTA licence’, and ‘your capacity to carry out the work’ were reported by one FD (33%). None agreed with ‘the legal responsibility you would be required to take on under the HTA regulations’. When asked if they there was anything which would have supported them to participate, ‘further training on how to implement the programme’, ‘additional support on the HTA licence’, and ‘financial compensation’ were reported by one FD (33%). None agreed with ‘individual review of your licence paperwork’ or ‘buddying with an FD already involved’.

The proportion of families giving consent to participate fluctuated, with an overall reduction over the course of the pilot (Table 1). Where reasons for refusing consent were reported, these included feeling that COVID‐19 testing was irrelevant, cultural reasons, and preferring to leave the deceased alone. In some cases, the relatives in ‘qualifying relationships’ could not agree on whether to consent. Reasons for giving consent were rarely documented.

The HTA reported that the overall experience of working on the project was positive. Despite requiring significant time and resource, they welcomed the opportunity to scrutinise the licensing framework and develop a working relationship with UKHSA. In processing licence applications, HTA staff had one‐to‐one discussions with FDs. They reported that the main barrier for FDs was that the sector had little previous experience of working under regulations, and had therefore not anticipated the requirements for detailed protocols for work related, but not specific, to the post‐mortem pilot, e.g. cleaning, security, and information governance.

### 3.3 Data Quality

Completeness of data submitted varied between FDs. Throughout the pilot, one FD did not submit a log containing details on eligibility assessments and seeking consent, but submitted the total number of funerals conducted. The majority of deceased individuals did not progress through the system as their eligibility for participation was either not assessed or not documented (Figure 2). Of those who were assessed as ineligible, the majority were hospital deaths.

To be eligible, a deceased individual needed to have been swabbed within 7 days of death. The median time from death to swabbing was 2 days (range 0 to 6).

**FIGURE 3:**
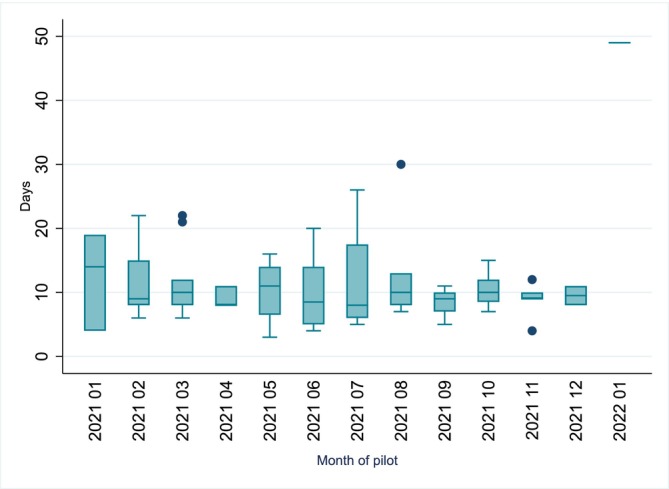
Box plot showing time (days) from death to report of SARS‐CoV‐2 result by month of pilot. Within each box, the lower limit, middle horizontal line, and upper limit show the 25th, 50th, and 75th percentiles. Outer bars show the lower and upper adjacent values; dots show outliers values.

The median time from death to SARS‐CoV‐2 result was 9 days (Figure 3). Time to test the specimens varied according to when it was received in the laboratory, as tests performed for surveillance rather than diagnostic purposes were batch‐reported.

### 3.4 Simplicity

FDs reported implementing the programme to be simple, but that the volume of work was too high. Feedback early in the implementation period was that the process for obtaining and documenting consent was not clear, which led to a change in the standard operating procedure (SOP). There was some resistance to digital data collection, which was complicated by inconsistent access to computers and Internet in different working areas within the FD premises.

### 3.5 Flexibility

The pilot was flexible where required. The time from death to specimen was increased in line with advice on feasibility from FDs, and again following analysis of post‐mortem data [5]. Updates were made to the information given to relatives of the deceased to reflect the changing public perception of the pandemic.

### 3.6 Usefulness

Linkage to the national COVID‐19 mortality dataset showed that three positive post‐mortem results could not be matched to a death which occurred within 28 days of a positive ante‐mortem SARS‐CoV‐2 test, and one could not be matched to a death within 60 days of a positive result. That individual had not been identified previously as a COVID‐19‐related death. This demonstrated a potential usefulness in identifying ‘missed’ COVID‐19 deaths, if the programme could be implemented at scale.

## 4 Discussion

The pilot showed post‐mortem surveillance using FDs was, in principle, feasible during the COVID‐19 pandemic. However, the pilot depended on the participation of FDs, which is not guaranteed throughout the sector or outside of a pandemic, and on the support of the HTA, which was specific to the pandemic context.

Participating FDs were not representative of the funeral sector. Smaller FDs (those not part of larger chains) appear to have found it easier to apply for a licence and implement the programme. The main barrier for FDs who initially expressed an interest, but who did not go on to join the programme, was capacity for participation. Staffing shortages prohibited some from taking on additional work, and some reported that the regulatory requirements were too onerous. Although only one FD reported financial support would have encouraged them to participate, this could have been a factor for other FDs who felt they lacked resource to participate and who did not respond to the questionnaire.

Relatives' willingness to consent waned over the pilot period. This was likely related to an overall reduction in COVID‐19‐related mortality and the vaccine roll‐out and may have been a result of ‘pandemic fatigue’ [20]. These may have contributed to the perception that the benefits of being involved (receiving the deceased individual's result and contributing to scientific understanding) no longer outweighed the disadvantages (disturbing the deceased and making decisions while coping with bereavement). Higher consent rates earlier in the pilot suggest that there could be circumstances when the proportion consenting would be great enough to yield a useful number of results. However, reasons for denying consent were rarely given and were not elaborated on; it is difficult to fully understand the motivations for and against participation.

The pilot system was necessarily voluntary, as gaining consent from a living relative is a legal requirement for taking human tissue samples in this context. A similar system implemented in different settings might yield different sampling rates. For example, a system implemented through hospital mortuaries would still require a relative's consent, but barriers to the site taking part would be reduced, as mortuaries are already subject to HTA regulations. A system implemented through coroners' examinations would depend on individual coroners considering post‐mortem testing to be appropriate, rather than relying on a relative's consent. Deceased individuals whose deaths are managed through hospital mortuaries and the coronial system are likely to be different to those whose deaths are managed through FDs; this would need to be explored and accounted for in implementing a post‐mortem surveillance system in a new setting.

Data progressed through the system more quickly than anticipated. Despite batch‐reporting, the median time from death to receipt of test result was 9 days. This was relevant as UK policy for most of the pilot was that COVID‐19 contacts should self‐isolate for 14 days [21]. This may have meant that it was plausible, and therefore necessary, to contact trace the deceased, as there was a chance that individuals would need to self‐isolate.

The pilot's aim was to demonstrate feasibility, not usefulness. Though the number of cases captured by the system was small, linkage to the mortality dataset demonstrated that the programme was capable of detecting individuals who died while SARS‐CoV‐2 positive, including those who were not previously known to be positive. However, testing alone cannot indicate whether the death was due to COVID‐19, and a positive result does not necessarily indicate active infection.

The programme was initially proposed in May 2020, early in the COVID‐19 pandemic. Community testing was restricted, overall mortality was increasing, and less was known about the disease. The pilot was implemented considerably later, following significant lead time for consultation and fulfilment of legal requirements. The feasibility and acceptability of the programme at a time of limited testing could therefore not be fully assessed, as community testing policy had changed and become more accessible by the time the pilot began.

Literature on post‐mortem surveillance for respiratory pathogens is limited. We could not identify other programmes where testing was carried out in the funeral sector or in the community. The validity of using post‐mortem URT samples is supported by studies undertaken in Spain, Germany, and Zambia [7, 8, 9]. However, the feasibility of conducting such a system routinely in the community has not been reported on.

No deceased individuals tested positive for other respiratory pathogens including influenza. The background rate of influenza was relatively low during the pilot period, so this was to be expected.

## 5 Conclusion

The pilot programme was sufficiently simple and flexible. We found post‐mortem surveillance to be feasible in theory, in that it was possible to identify those who died whilst SARS‐CoV‐2 positive and who had not been identified through other routes. However, despite being acceptable in principle, attempts to increase the scale of the programme were largely ineffective. Two key barriers to this were identified: sufficient FD participation and sufficient relative consent rate.

Some of the barriers to FD participation could be addressed through embedding the surveillance system outside of a pandemic, when staffing and resource is less likely to be under pressure. However, it is likely that some FDs' willingness to participate stemmed from wanting to contribute to the pandemic response.

The benefit of implementing post‐mortem surveillance in FDs is that it provides an opportunity to test individuals who die outside of hospital and are therefore unlikely to have had recent ante‐mortem testing. Alternative processes which may capture community deaths include liaising with coroners, to whom unexplained deaths are referred, and working with hospital mortuaries, which manage some community deaths. Although deaths managed in these systems are likely to be different to those managed by FDs, both of these sectors are better placed to work within the regulatory framework set out in the Human Tissue Act, which may enable greater participation.

One such approach would be a system in which pathologists conducting coroner's post‐mortem examinations are encouraged to submit URT samples to be tested for a range of respiratory viruses. Deaths of particular interest would include community and unexpected deaths, including where a non‐infectious cause of death is identified. This would necessarily be a voluntary surveillance system as coroners work independently to establish a cause of death. However, it may be a more acceptable system in that URT sampling would not require significant additional training or resource.

The development and implementation of the pilot were time intensive. Future programmes would be less so now that processes are established, but lead time for implementing a system anew should not be underestimated. The considerable time taken to consult on the programme and establish a protocol compliant with regulations meant that the pilot could not be implemented at a time of the pandemic when community testing was low, as had been initially intended.

A post‐mortem surveillance system such as this would be most useful when there is little information from other sources, that is, when other forms of testing are limited. The system could only be made useful if implemented at scale, which is only possible when families are most willing to consent to swabbing. The pilot suggested that this was at times when cases and mortality were high and community testing relatively limited, though we were unable to test the pilot at a time when testing was highly restricted. These findings may not be generalisable to other respiratory pathogens, or other diseases, as the public perception of the threat they pose will be different. Further work is needed to understand how frequently consent would be given outside of a pandemic context.

Despite the small size of this pilot, we were able to detect SARS‐CoV‐2 positive deaths which had not been ascertained through community testing. We recommend that implementation of post‐mortem surveillance for respiratory pathogens be considered. However, we suggest using alternative settings to FDs because of the regulatory and resource burden.

## Author Contributions

All authors contributed to the development and evaluation of the pilot system. HE, JH, and GD conceptualised the system; AS and MZ advised on laboratory testing; HE, JH, HW, and AI were responsible for implementation and data collection and analysis; HE evaluated the system and prepared the manuscript; all authors contributed to and reviewed the manuscript.

## Ethics Statement

Ethical approval for the pilot surveillance programme was obtained from the Public Health England Research, Ethics, and Governance Group on 28 July 2020 (reference: NR0222).

## Consent

No living patients were involved in the pilot surveillance programme. Consent for obtaining specimens from deceased individuals was sought from the person in the highest available ‘qualifying relationship’ to the deceased, in accordance with the Human Tissue Act 2004.

## Conflicts of Interest

The authors declare no conflicts of interest.

## Permission to Reproduce Material From Other Sources

Not applicable.

## Data Availability

This work is carried out under Regulation 3 of The Health Service (Control of Patient Information) (Secretary of State for Health, 2002). Data cannot be made publicly available for ethical and legal reasons, i.e. public availability would compromise patient confidentiality as data tables list single counts of individuals rather than aggregated data.

## Endnotes

^1^
Section 16 [2](c) – the removal from the body of a deceased person (otherwise than in the course of an anatomical examination or a post‐mortem examination) of relevant material of which the body consists or which it contains, for use for a scheduled purpose other than transplantation.

^2^
In England, a death is reported to the coroner if: A doctor did not treat the person during their last illness, a doctor did not see or treat the person for the condition from which they died within 28 days of their death, or the death was sudden, unexpected, or unnatural.
